# Quantifying tumor-infiltrating immune cells from transcriptomics data

**DOI:** 10.1007/s00262-018-2150-z

**Published:** 2018-03-14

**Authors:** Francesca Finotello, Zlatko Trajanoski

**Affiliations:** Biocenter, Division for Bioinformatics, Medical University of Innsbruck, Innrain 80, 6020 Innsbruck, Austria

**Keywords:** TILs, RNA-seq, Next-generation sequencing, NGS, Deconvolution, Gene expression

## Abstract

By exerting pro- and anti-tumorigenic actions, tumor-infiltrating immune cells can profoundly influence tumor progression, as well as the success of anti-cancer therapies. Therefore, the quantification of tumor-infiltrating immune cells holds the promise to unveil the multi-faceted role of the immune system in human cancers and its involvement in tumor escape mechanisms and response to therapy. Tumor-infiltrating immune cells can be quantified from RNA sequencing data of human tumors using bioinformatics approaches. In this review, we describe state-of-the-art computational methods for the quantification of immune cells from transcriptomics data and discuss the open challenges that must be addressed to accurately quantify immune infiltrates from RNA sequencing data of human bulk tumors.

**Electronic supplementary material:**

The online version of this article (10.1007/s00262-018-2150-z) contains supplementary material, which is available to authorized users.

## Introduction

Tumors are not merely masses of malignant cells, but complex ecosystems composed of different types of cells. Among these cells, tumor-infiltrating immune cells play a central role in tumor control and response to therapy [1, 2]. For instance, cytotoxic CD8^+^ T cells are the primary effectors of anticancer immunity, as they can specifically recognize and kill tumor cells bearing neoantigens (i.e., tumor-specific antigens arisen from the expression of mutated genes) [3]. But immune cells can also exert immunosuppressive functions supporting tumorigenesis and immune evasion, as in the case of regulatory T (T_reg_) cells [4].

Therefore, the quantification of the different types of tumor-infiltrating immune cells can shed light on the mechanisms underlying the anticancer immune response and might help to assess the immunogenic effects of anticancer therapies, ultimately guiding the rational design of combination therapies. Most importantly, provided that immunotherapy with immune checkpoint blockers is only effective in a limited fraction of patients [5], the quantification of the immune infiltrates in pre- and on-treatment tumor samples holds promise to identify novel biomarkers for the monitoring and prediction of response.

So far, the composition of the immune infiltrates of human cancers has been investigated mainly with immunohistochemistry (IHC), immune fluorescence (IF), and flow cytometry. Now that the steep decrease in costs of next-generation sequencing (NGS) technologies [6] has motivated its application to routine oncology and has fostered large-scale collaborative efforts like the cancer genome atlas (TCGA) [7], we are gaining access to an unprecedented amount of RNA sequencing (RNA-seq) data describing the tumor microenvironment. The composition of tumor-infiltrating immune cells can be characterized from bulk tumor RNA-seq data using computational approaches based on a set of immune-specific marker genes or expression signatures.

**Fig. 1:**
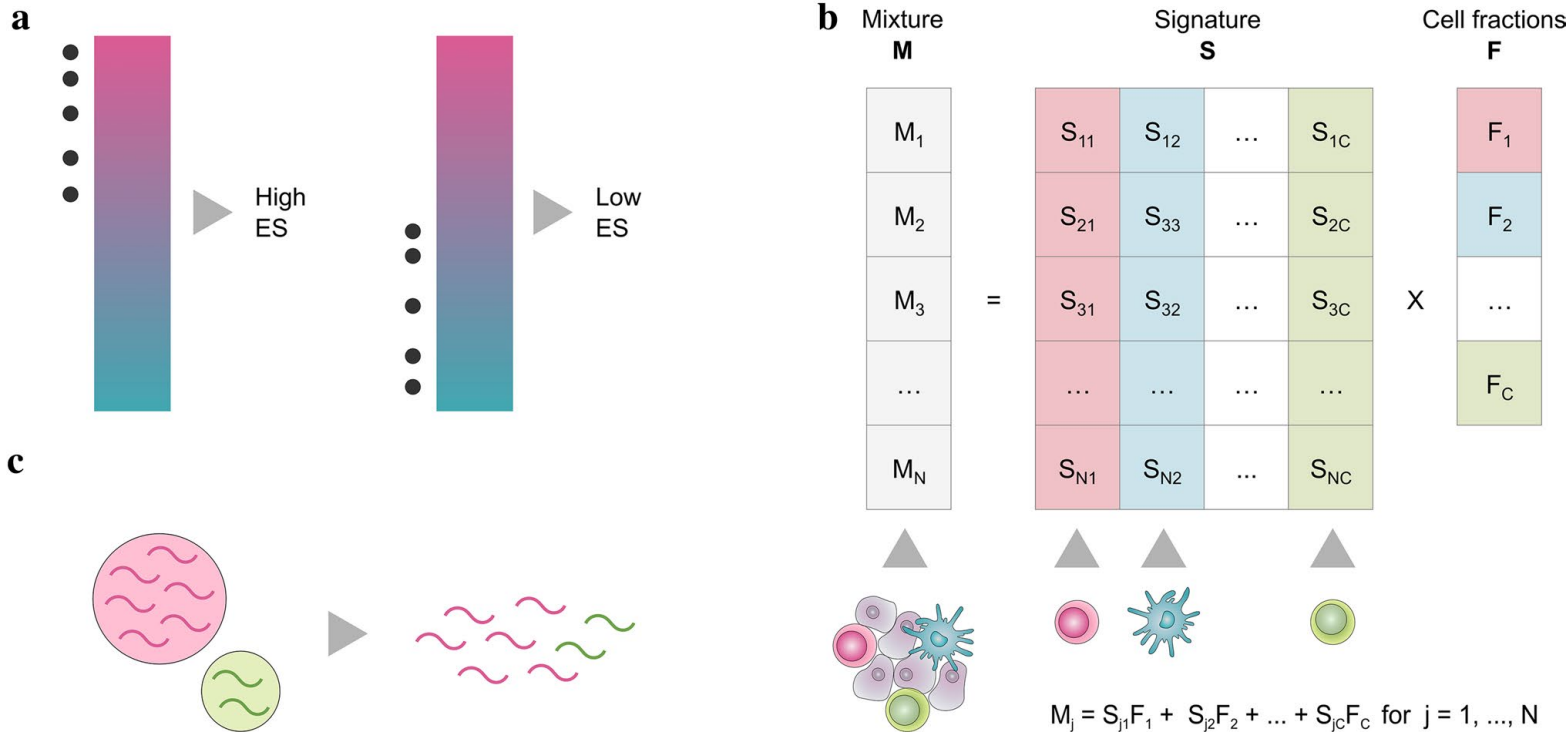
**a** Approaches based on gene set enrichment analysis rank the genes according to their expression in a sample and compute an enrichment score (ES) considering the position of a set of cell-type-specific marker genes (grey dots) in the ranked list. The ES is high when the marker genes are among the top highly expressed genes (magenta) and low otherwise (cyan). **b** Deconvolution algorithms model the expression of a gene in a mixture **M** as a linear combination of the expression of that gene in the different cell types, whose average expression profiles are summarized in a signature matrix **S**, weighted by the relative fractions **F** of the cell types in the mixture. **c** Cell types with higher amount of total mRNA contribute more to the cumulative expression of a heterogeneous sample and might be overestimated by deconvolution methods

The most famous approach for the analysis of maker genes is gene set enrichment analysis (GSEA) [8]. GSEA-based methods compute an enrichment score (ES) that is high when the genes specific for a certain cell type are amongst the top highly expressed in the sample of interest (i.e., the cell type is enriched in the sample) and low otherwise (Fig. 1a).

Unlike GSEA-based approaches that can only compute a semi-quantitative score describing the enrichment of a cell type in a sample, deconvolution methods can quantitatively estimate the relative fractions of the cell types of interest. Deconvolution algorithms consider gene expression profiles of a heterogeneous sample as the *convolution* of the gene expression levels of the different cells, and estimate the unknown cell fractions leveraging on a signature matrix describing the cell-type-specific expression profiles (Fig. 1b).

In this review, we describe state-of-the-art computational methods that quantify immune cells from expression data of cell mixtures using marker genes coupled with GSEA or other scoring approaches, or leveraging on deconvolution algorithms and immune cell expression signatures (Table 1). Finally, we discuss the issues and open challenges that must be addressed to accurately quantify immune infiltrates from bulk tumor RNA-seq data.

**Table 1:** Features of the computational tools for the quantification of tumor-infiltrating immune cells from transcriptomics data considered in this review: tool or function name, algorithm type (M = marker genes, P = partial deconvolution, C = complete deconvolution), main method, cell types quantified using the embedded gene sets or signature profiles, code availability, name of the method in the CellMix package [9], reference publication

Tool	Type	Method	Cell types	Code availability	CellMix	References
TIminer	M	PrerankedGSEA	Different gene sets with 31 [10], 28 [11], and 64 cell types [12]	http://icbi.i-med.ac.at/software/timiner/timiner.shtml (Docker image)		[13]
xCell	M	ssGSEA	64 immune and non-immune cell types	http://xcell.ucsf.edu/ (R script, web tool)		[12]
MCP-counter	M	Geometric mean of expression of marker genes	8 immune cells, fibroblasts, and endothelial cells	http://github.com/ebecht/MCPcounter (R script)		[14]
–	P	Linear least squares regression	17 immune cell types		lsfit	[15]
–	P	Constrained least square regression	–		qprog	[16]
DeconRNASeq	P	Constrained least square regression	–	DeconRNASeq package available on Bioconductor (R package)		[17]
PERT	P	Non-negative maximum likelihood		Supplementary material in the original publication (Octave)		[18]
CIBERSORT	P	Nu support vector regression	22 immune cell types	https://cibersort.stanford.edu/ (R script, java executable, web tool)		[19]
TIMER	P	Linear least square regression	6 immune cell types	https://cistrome.shinyapps.io/timer/ (web tool)		[20]
EPIC	P	Constrained least square regression	6 immune cell types, fibroblasts, endothelial cells, and uncharacterized cells	https://gfellerlab.shinyapps.io/EPIC_1-1 (R script, web-interface)		[21]
quanTIseq	P	Constrained least square regression	10 immune cell types, uncharacterized cells	http://icbi.i-med.ac.at/software/quantiseq/doc/index.html (Docker image)		[22]
deconf	C	Non-negative matrix factorization	-	Supplementary material in the original publication (R package)	deconf	[23]
ssKL	C	Non-negative matrix factorization	–		ssKL	[24]
ssFrobenius	C	Non-negative matrix factorization	–		ssFrobenius	[25]
DSA	C	Quadratic programming	–	https://github.com/zhandong/DSA (R package)	dsa	[26]
MMAD	C	Maximum likelihood over the residual sum of squares	–	http://sourceforge.net/projects/mmad/ (Matlab)		[27]

## Gene set enrichment analysis and other scoring methods based on marker genes

The original GSEA approach determines whether an a priori defined set of genes shows statistically significant differences between two biological conditions or states [8]. In brief, the genes in the expression data set are ranked considering their correlation with the condition of interest. Then, for each position in the ranked list, a running-sum statistic is increased when one of the genes belonging to the query set is encountered and decreased otherwise. Finally, the ES is computed as the maximum deviation from zero of the running-sum statistic.

In the characterization of tumor-infiltrating lymphocytes from expression data, the pre-ranked version of the GSEA algorithm (*GSEAPreranked*) can be used to compute sample-specific ES. Briefly, gene ranks are calculated for single samples considering their (normalized) expression and ES are computed similarly to GSEA, but considering gene ranks instead of correlations. Using this approach, Angelova et al. defined 31 custom gene sets representing genes up-regulated in specific immune cell sub-populations and used GSEAPreranked to characterize tumor-infiltrating immune cells in colorectal cancer (CRC) patients [10]. This approach was later extended through the definition of 28 pan-cancer immune gene sets and used to analyze more than 8000 samples across 19 different TCGA solid cancers (results available at https://tcia.at/) [11].

These approaches were recently integrated in TIminer, a user-friendly, computational framework to perform different onco-immuno-genomic analyses, including: human leukocyte antigens (HLA) typing, neoantigen prediction, determination of tumor immunogenicity, and quantification of tumor-infiltrating immune subsets with GSEAPreranked analysis based on three different immune gene set compendia [13].

An alternative approach is single-sample GSEA (ssGSEA), which computes an ES representing the degree to which genes in a particular gene set are coordinately up- or down-regulated within a single sample [28]. With respect to the original GSEA framework, ssGSEA ranks the genes by their absolute expression in a sample and computes ES by integrating the differences between the empirical cumulative distribution functions of the gene ranks.

xCell is a recently published method based on ssGSEA that estimates the abundance scores of 64 immune cell types, including adaptive and innate immune cells, hematopoietic progenitors, epithelial cells, and extracellular matrix cells [12]. xCell is based on a novel compendium of 489 gene sets extracted from large-scale expression data from different projects and studies: FANTOM5 [29], ENCODE [30], Blueprint [31], Immune Response In Silico (IRIS) [32], Human Primary Cell Atlas (HPCA) [33], and Novershtern et al. [34]. For each cell type, the xCell abundance scores are computed through four main steps: (i) ssGSEA performed independently for each of the 489 gene sets using the GSVA R package [35]; (ii) averaging of the ES across all gene sets belonging to a cell type; (iii) platform-specific conversion of ES into abundance scores; and, (iv) corrections of correlations between closely related cell types using a “spillover” approach similar to that used for flow cytometry data analysis. Although the final xCell abundance scores cannot be directly interpreted as cell fractions, they showed high correlation with the true cell proportions [12].

More recently, Becht et al. developed MCP-counter, a method for the quantification of tumor-infiltrating immune cells, fibroblasts, and epithelial cells based on a stringent and robust set of marker genes [14]. For each cell type and sample, the abundance score is computed as the geometric mean of the expression values of cell-type-specific genes. Since the scores are expressed in arbitrary units, they cannot be directly interpreted as cell fractions, nor compared between cell types. However, quantitative validation using well-defined cell mixtures showed high correlation between the estimated scores and the true cell fractions, proving the value of MCP-counter for inter-sample comparison. To demonstrate the prognostic value of these estimates, MCP-counter has been used to quantify immune and non-immune cells in more than 19,000 samples across 32 non-hematological tumors [14].

Additional set of immune cell marker genes are available in CellMix, an R package that provides a standardized and user-friendly interface for accessing different deconvolution algorithms, expression signatures, sets of marker genes, as well as benchmark data sets derived from the literature [9].

## Deconvolution of cell mixtures using expression signatures

The deconvolution problem can be formulated as a system of equations that describe the expression of each gene in a heterogeneous sample as a linear combination of the expression levels of that gene across the different cell subsets present in the sample, weighted by their relative cell fractions (Fig. 1b). Although the relationship between the expression levels of pure and heterogeneous samples is not strictly linear, previous work has shown that the linearity assumption is reasonable [36].

Abbas et al. proposed an approach based on linear least square regression to solve the deconvolution problem and, then, force all negative estimates to zero and re-normalize the cell fractions to sum up to one [15]. To test the method on the deconvolution of immune cell fractions from microarray expression data, they built a signature matrix spanning 17 blood-derived immune cell subsets profiled by the IRIS project. The approach was validated on mixtures of transformed immune cell lines, as well as of blood-derived immune cells from patients affected by systemic lupus erythematous, using flow cytometry or Coulter counter as gold standard technology, respectively. The benchmarking proved high correlation between the true and the estimated cell fractions, although only a limited subset of the considered cell types was assessed [15].

Gong et al. used constrained least squares and quadratic programming to identify the deconvolution solution with the lowest error while simultaneously forcing the cell fractions to be non-negative and to sum up to one [16]. The algorithm was tested on the deconvolution of whole-blood samples from multiple sclerosis patients using microarray-based signatures from [15] (excluding neutrophils), obtaining a high correlation with flow cytometry cell fractions [16]. The constrained-regression framework was then adapted by Gong and Szustakowski for the analysis of RNA-seq data and implemented in an R package called DeconRNASeq [17]. The algorithm was validated on simulated data generated by mixing RNA-seq data from five human tissues (brain, skeletal muscle, lung, liver, and heart) [37] and leveraging on a signature matrix built from RNA-seq data of the Illumina’s Human Body Map 2.0 project. Although no novel immune signatures were developed, the tool can be coupled, in principle, with any signature matrix.

Qiao et al. proposed a perturbation model, PERT, to account for variability in gene expression due to different microenvironmental and developmental conditions [18]. PERT tackles the deconvolution problem using non-negative least squares and simultaneously perturbs the signature profiles to capture the transcriptional variations in the mixture data with respect to the reference profiles. Compared to simple non-negative least squares, PERT approach coupled with a signature matrix derived from [34] showed superior performance in the deconvolution of microarray data from uncultured mononucleated and Lin^−^ umbilical cord blood samples [18]. PERT also outperformed two approaches, NNML and NNML_np_ [18], based on non-negative maximum likelihood (NNML) models and on latent Dirichlet allocation (LDA) [38].

The recently developed CIBERSORT algorithm considers a signature matrix built from microarray data, which describes the expression fingerprints of 22 immune cell phenotypes, including different cell types and functional states [19]. CIBERSORT estimates the cell fractions using nu support vector regression (*ν*-SVR). For each sample, *ν*-SVR is run with three different *ν* values (0.25, 0.5, and 0.75) and the solution providing the lowest root-mean-square error (RMSE) between the true expression and the estimated expression $\hat {M}=S \times \hat {F}$ is selected. Also in this approach, the coefficients are forced to non-negative values and normalized to sum up to one. Validated on microarray data of cell mixtures derived from blood and from lymph node biopsies, CIBERSORT proved to have a high accuracy in the simultaneous deconvolution of nine and three immune cell subsets, respectively, whereas it showed a lower accuracy in the quantification of gamma-delta T cells [19]. Tested on simulated mixtures of four malignant immune cell types, it also proved robustness to various levels of noise and unknown tumor content. CIBERSORT was applied to about 18,000 microarray data sets across 39 solid and hematological cancers (results available at https://precog.stanford.edu/) [39].

Li et al. developed a multi-step computational approach, TIMER, to estimate the abundances of six immune cell types in 32 cancer types leveraging on a list of immune-specific markers derived from the IRIS database and on immune cell expression signatures extracted from the HPCA microarray data [20]. Each cancer expression matrix under investigation, derived from RNA-seq or microarray data, is merged with the immune cell expression matrix and normalized with Combat [40] to remove batch effects. Signature genes are identified separately for each cancer type by selecting from the immune cell markers the genes that are negatively associated with tumor purity. Finally, for each cancer type, the signature matrix is built from the normalized immune cell profiles considering the selected immune cell markers. TIMER performs deconvolution using the linear least square regression approach proposed in [15] and forces all negative estimates to zeros. The estimation is repeated several times with an increasingly smaller set of T-cell markers to reduce the correlation between the estimated CD8^+^ and CD4^+^ T cell proportions. Unlike CIBERSORT, the final estimates are not normalized to sum up to one and, thus, cannot be neither interpreted directly as cell fractions [41] nor compared across different immune cell types and data sets [20]. TIMER was validated on simulated mixtures, as well as on TCGA samples considering as ground truth quantized neutrophil abundances estimated from images of hematoxylin and eosin (H&E)-stained tissue slides and lymphocytic infiltration scores computed from DNA methylation data [20]. TIMER was applied to more than 10,000 samples across 32 cancer types of TCGA (results available at https://cistrome.shinyapps.io/timer/) [42].

Racle et al. recently developed a tool to Estimate the Proportion of Immune and Cancer cells (EPIC) [21]. EPIC uses constrained least square regression to explicitly incorporate the non-negativity constraint into the deconvolution problem and to impose that the sum of all cell fractions in each sample does not exceed one. The difference between one (i.e., 100% of the cells in the mixture) and the sum of the deconvoluted cell fractions represents the proportion of uncharacterized cells in the mixture that are not accounted by the signature matrix used for deconvolution and, in RNA-seq data from bulk tumors, represents the tumor content [21]. EPIC can be run using two RNA-seq-based signature matrices which describe the expression signatures of: (i) six blood-circulating immune cell types, or (ii) five tumor-infiltrating immune cell types plus endothelial cells and cancer-associated fibroblasts (CAF), whose expression signatures were extracted from melanoma single-cell RNA-seq data [43]. After validation on literature data, EPIC was further tested on RNA-seq data from lymph nodes collected from four melanoma patients. EPIC estimates showed a high agreement with the cell fractions computed with flow cytometry for both the immune and the uncharacterized cells [21].

quanTIseq is, currently, the most recent deconvolution tool and is specifically developed for RNA-seq data [22]. It is based on constrained least square regression (to consider the non-negativity and sum-to-one constraints) and on a novel signature matrix derived from a compendium of 51 RNA-seq data sets from purified or enriched immune cell types, including also T_reg_ cells and classically (M1) and alternatively (M2) activated macrophages. To avoid inconsistencies between the mixture and the signature matrix, quanTIseq implements a full pipeline for the analysis of RNA-seq data, from read pre-processing to deconvolution of cell fractions. Moreover, quanTIseq allows complementing the deconvolution output with data from H&E images to perform “in silico multiplexed immunodetection”, namely to obtain cell density estimates (i.e., cells per mm^2^) for all the considered cell types. quanTIseq obtained a high deconvolution performance not only on literature data sets, blood-derived immune cell mixtures and simulated data, but also on tumor RNA-seq data from different cancer types, and has been used to quantify the immune cell fractions in more than 8000 TCGA bulk tumors profiled with RNA-seq (results available at https://tcia.at) [22].

Although this review focuses on methods for the analysis of human data, it is worth mentioning that bioinformatics tools for quantifying and comparing the abundances of immune cells in mice samples [44–46] or to infer immune cell composition in human tissues leveraging on mouse expression data [47, 48] are also available.

## Simultaneous deconvolution of cell fractions and expression profiles

Deconvolution algorithms described in the previous paragraph are called “partial”, as opposed to “complete” deconvolution methods, which estimate relative cell fractions and simultaneously disentangle their expression profiles. Starting from the pioneering work of Venet et al. [49], several methods have leveraged on non-negative matrix factorization (NMF) to alternate least-square estimation of the cell proportions and expression profiles [23, 50, 51].

However, NMF is a completely unsupervised approach and, thus, it might decompose the mixture matrix into components that are not related to the cell types of interest. By using well-defined mixtures of four hematological cancer cell lines, Gaujoux et al. demonstrated that the incorporation of prior knowledge from cell-specific marker genes into NMF-based methods can dramatically improve the results of complete deconvolution [51]. All semi-supervised NMF approaches tested by Gaujoux et al. (deconf [23], ssKL [24], and ssFrobenius [25]), are implemented in the CellMix R package [9].

DSA is a complete deconvolution algorithm that uses quadratic programming to infer the cell fractions and the expression profiles in complex tissues leveraging on a set of marker genes that are highly expressed in specific cell types [26]. Tested on microarray data from mixtures of three malignant immune cell lines, the algorithm faithfully reconstructed the true cell fractions and expression profiles [26]. Validation on simulated data from mixtures of six different immune cell types showed high correlation between the estimated and true expression profiles, except for naïve B cells and basophils [26].

MMAD is a deconvolution algorithm that can perform both partial deconvolution, when the cell proportions or the signature profiles are known, or complete deconvolution based on marker genes [27]. In case marker genes are not known a priori, MMAD identifies cell-specific genes using k-means clustering of the most variable genes in the data set. Tested on mixture expression data from four hematological cancer cell lines, MMAD accurately inferred the unknown cell fractions without relying on known marker genes [27]. Moreover, accurate reconstruction of the constituent expression profiles was demonstrated using simulated data and experimental mouse expression data [27].

Complete deconvolution methods are complex but promising tools for the in silico dissection of tissues and cell mixtures from expression data when a priori knowledge on cell-specific signatures is not available. However, to be applied for the immuno-phenotyping of bulk tumors, their performance in the quantification of a higher number of immune cell types form bulk tumor data characterized by variable levels of noise and unknown content must be thoroughly assessed.

Deconvolution algorithms that estimate both cell-type proportions and expression profiles can be valuable also when the contribution of the healthy cells needs to be subtracted from bulk expression data to characterize the pure tumor molecular fingerprints (i.e., when stroma, healthy tissue, and tumor-infiltrating immune cells are considered a confounding factor). The extraction of tumor expression signatures from heterogeneous samples is important to guide patient treatment, for instance through the identification of cancer subtypes and tracking of the site of origin. Computational methods like ISOpure [52, 53] and DeMix [54] can be used to simultaneously quantify tumor purity and disentangle tumor-specific expression signatures from bulk tumor expression data. A comparative benchmarking on controlled, heterogeneous samples revealed a superior performance of DeMix compared to ISOpure in the deconvolution of RNA-seq data from mixtures of two lung cancer cell lines [55].

## Challenges in the quantification of tumor-infiltrating immune cells from RNA sequencing data

When dealing with bulk tumors, the first desired feature of a deconvolution algorithm is the robustness to the unknown tumor content, which usually accounts for the largest fraction of cells in the sample. Although the expression profiles of tumor cells are usually not accounted for by the signature matrix, they contribute largely to the cumulative expression of the bulk tumor sample.

Tested on simulated microarray data characterized by different tumor contents, CIBERSORT demonstrated robust deconvolution performance [19]. The NNML_np_ approach proposed by Quiao et al. infers cell fractions assuming the presence in the mixture of an additional, unknown population not described in the signature matrix used for deconvolution [18].

EPIC and quanTIseq use non-negative least squares to allow the sum of cell proportions for the subsets considered by the signature matrix to be lower than one, thus estimating the fraction of uncharacterized cells [21, 22]. EPIC showed superior performance to ISOpure in the prediction of cancer cell fractions from RNA-seq data [21], whereas quanTIseq demonstrated high accuracy in the quantification of the unknown tumor content in 1700 simulated data sets from bulk tumor RNA-seq [22]. Besides being robust to the unknown tumor content, these approaches quantify the immune cell fractions referred to the total bulk tissue, allowing both intra- and inter-sample comparison; the latter is not guaranteed, instead, when cell proportions are referred only to the screened immune cell types [56].

Another challenge for deconvolution algorithms is due to *multicollinearity*, i.e., to the high correlation of the expression profiles of closely related cell types. This issue is exacerbated when the gene expression levels in low-abundance cell types are masked by the expression of the same gene(s) in a more abundant cell subset (i.e., *signal dilution* [36]).

CIBERSORT simultaneously estimates the proportion of 22 different cell types and phenotypes leveraging on feature selection performed by the *ν*-SVR framework [19]. Whether regularization approaches like those embedded in SVR are sufficient to overcome multicollinearity in regression-based deconvolution is still an object of debate [41, 57, 58].

TIMER limits the quantification to six immune cell types and performs several runs of deconvolution of the CD8^+^ and CD4^+^ T cell proportions to iteratively decrease their correlation [20]. quanTIseq uses an heuristic approach to disentangle the fractions of T_reg_ cells and non-regulatory CD4^+^ T cells: a consensus estimation of the T_reg_ cells is derived by running deconvolution twice, once with the full signature matrix and once excluding the CD4^+^ T cell signature [22]. EPIC and MCP-counter, although more limited in the number of screened immune cell types with respect to CIBERSORT and quanTISeq (see a list of the cell types quantified by the reviewed approaches in Supplementary Table 1), they allow the quantification of non-immune cell types like CAFs and epithelial cells [14, 21].

Albeit, in principle, GSEA-based approaches should benefit by the possibility of quantifying each cell subset individually, and the use of gene lists hampers the distinction of closely related subtypes.

Finally, besides the technical challenges posed by multicollinearity, deep deconvolution is further hampered by the intrinsically plastic and dynamic nature of the immune system, which results in the co-existence of a continuum of immune phenotypes and prevents a clear distinction between the concepts of *cell type* and *cell state* [59].

RNA-seq is currently the reference technology for transcriptome-wide quantification of gene expression [60]. The “digital” nature and the wider dynamic range of RNA-seq data compared to microarrays have fostered the development of novel bioinformatics tools and statistical models [60]. Similarly, the deconvolution of RNA-seq data requires dedicated methods or re-adaptation of tools designed for microarray data.

We have previously proposed a model to transform RNA-seq data to be analyzed, CIBERSORT, which was originally developed and validated using only microarray data. Specifically, we considered tumor samples from three TCGA cancers for which both microarrays and RNA-seq data were available and estimated a gene-specific model by fitting a smoothing spline with four degrees of freedom to transform RNA-seq data, as log-transformed transcripts per millions (TPM), into “microarrays-like” data [11]. We then used the model to transform RNA-seq data from more than 8000 TCGA tumors across 19 different cancer types and inferred the fractions of tumor-infiltrating immune cells with CIBERSORT (results available at https://tcia.at) [11]. Similarly, Ali et al. [61] analyzed with CIBERSORT more than 11,000 breast tumor RNA-seq data sets normalized with limma voom, a method that transforms RNA-seq log counts to enable downstream application of microarray-specific methodologies [62].

So far, EPIC and quanTIseq are the only methods specifically developed for RNA-seq data. quanTIseq, in particular, implements a full pipeline for the analysis of RNA-seq data, which includes: (i) read pre-processing; (ii) quantification of gene expression; and (iii) expression normalization, gene re-annotation, and deconvolution of cell fractions and densities. The controlled handling of the analytical steps that forego deconvolution is of paramount importance because they can strongly affect deconvolution results by leading to inconsistencies between the mixture and the signature matrices or by reducing the linearity assumed for the input data [63, 64].

An intrinsic limitation of deconvolution methods based on linear regression is that they assume a Gaussian distribution of the input data, whereas un-normalized RNA-seq counts are more accurately described by a negative binomial distribution [60]. Although data normalization can ameliorate this issue [62], future deconvolution approaches might exploit methods that do not rely on data normality, like LDA models [18, 52].

As different cell types can have significantly different mRNA contents [65], expression mixtures and, consequently, deconvolution results can be biased toward cell types characterized by a higher mRNA content (Fig. 1c). Although this bias is overlooked by several deconvolution methods, tools correcting for differences in mRNA content are available [21, 22, 27].

Finally, although many of the approaches presented in this review are intended for the analysis of expression data from various types of heterogeneous samples, the achievement of high accuracy in the deconvolution of tumor-infiltrating immune cells might require the development of approaches optimized for the tissue and disease context under investigation. For instance, as the transcriptional fingerprints of immune cells change depending on the microenvironment they reside in [4], EPIC uses two different signature matrices: one for the analysis of blood-derived cell mixtures and one for the analysis of bulk tumors [21]. However, despite some differences in the results obtained with the signature matrices defined from circulating or tumor-infiltrating immune cells, a clear pattern in the performance could not be identified [21]. Alternatively, perturbation models like that implemented in PERT allow accounting for differences from the signature expression profiles due to time- and context-dependent characteristics of the samples of interest [18]. Complete deconvolution methods offer even greater flexibility as they do not rely on reference expression profiles, but are inevitably limited by the higher complexity of the mathematical problem they aim to address.

Overall, to maximize their accuracy in tumor RNA-seq data analysis, deconvolution methods might need to be tailored for specific cancer entities to take into consideration the tissue and disease context, not only for extracting the expression signatures of tumor-infiltrating immune cells, but also to optimally select immune cell signature genes taking into account tumor-specific aberrant expression. In this regard, single-cell RNA-seq can help to reconstruct the expression fingerprints of the different cells of the tumor microenvironment [21, 66].

## Conclusions

The quantification of tumor-infiltrating immune cells has the potential to disentangle the multi-faceted role of the immune system in tumor control and response to therapy and, ultimately, to maximize the efficacy of anticancer therapies. This review portrays the currently available computational methods that can be used to quantify the immune infiltrates from bulk tumor RNA-seq data. More broadly, these algorithms can be valuable to dissect the cellular heterogeneity of different tissues and cell mixtures and can be applied to study other human diseases.

Currently, alternative technologies for the quantification of tumor-infiltrating immune cells like multiplexed IF or IHC [67] are within the reach only of specialized laboratories due to the high costs and complex experimental procedures they entail. Moreover, RNA-seq data generated from bulk tumor can be used to simultaneously extract, besides immune cell fractions, different immunological features relevant for cancer immunology like HLA types, T- and B-cell receptor repertoires, tumor neoantigens [68], and information about the cell functional orientation and state, including exhaustion or anergy.

Overall, deconvolution algorithms can be used to mine the vast amount of tumor RNA-seq data that is being generated in small-to-large-scale genomic projects and routine oncology—with more than 10,000 cases available through the NCI Genomic Data Commons portal alone (https://portal.gdc.cancer.gov, Data Release 8.0, August 22, 2017)—and might represent, in the near future, powerful tools for the opening of new avenues in personalized medicine.

### Electronic supplementary material

Below is the link to the electronic supplementary material.

Supplementary material 1 (PDF 50 KB)

## Abbreviations

CAF: Cancer-associated fibroblasts
CRC: Colorectal cancer
ES: Enrichment score
GSEA: Gene set enrichment analysis
H&E: Hematoxylin and eosin
HLA: Human leukocyte antigen
HPCA: Human primary cell atlas
IF: Immune fluorescence
IHC: Immunohistochemistry
IRIS: Immune response in silico
LDA: Latent Dirichlet allocation
M1: Classically activated macrophages
M2: Alternatively activated macrophages
mRNA: Messenger RNA
NGS: Next-generation sequencing
NMF: Non-negative matrix factorization
NNML: Non-negative maximum likelihood
RMSE: Root-mean-square error
RNA-seq: RNA sequencing
ssGSEA: Single-sample gene set enrichment analysis
SVR: Support vector regression
TCGA: The cancer genome atlas
TPM: Transcripts per millions
T_reg_: Regulatory T cells
