# Supplementary material for: Quantifying tumor-infiltrating immune cells from transcriptomics data
Source: Cancer Immunol Immunother. 2018 Mar 14;67(7):1031–40. doi: 10.1007/s00262-018-2150-z (PMC6006237; doi:10.1007/s00262-018-2150-z)
Supplement: Supplementary file 1 — Supplementary material 1 (PDF 50 KB) [file 262_2018_2150_MOESM1_ESM.pdf]

Supplementary Table 1: List of immune cell types quantified by the methods for which novel gene sets or expression signatures were made available in the original publication (full references in the main text).

Abbas et al.	Resting helper T cells, activated helper T cells, resting cytotoxic T cells, activated cytotoxic T cells, resting B cells, activated B cells, IgM memory B cells, IgA/IgG memory B cells, plasma cells, resting natural killer cells, activated natural killer cells, monocytes, activated monocytes, macrophages, resting dendritic cells, activated dendritic cells, neutrophils.
Newman et al.<br>CIBERSORT	Naïve $CD4^+$ T cells, resting memory $CD4^+$ T cells, activated memory $CD4^+$ T cells, follicular helper T cells, gamma-delta T cells, $CD4^+$ regulatory T cells, $CD8^+$ T cells, resting natural killer cells, activated natural killer cells, naïve B cells, memory B cells, plasma cells, monocytes, unstimulated (M0) macrophages, classically activated (M1) macrophages, alternatively activated (M2) macrophages, resting mast cells, activated mast cells, resting dendritic cells, activated dendritic cells, neutrophils, eosinophils.
Angelova et al.<br>TIminer	Activated B cells, activated $CD4^+$ T cells, activated $CD8^+$ T cells, central memory $CD4^+$ T cells, central memory $CD8^+$ T cells, effector memory $CD4^+$ T cells, effector memory $CD8^+$ T cells, cytotoxic cells, gamma-delta T cells, immature B cells, memory B cells, regulatory T cells, follicular helper T cells, type 1 helper T cells, type 17 helper T cells, type 2 helper T cells, $CD56^{bright}$ natural killer cells, $CD56^{dim}$ natural killer cells, eosinophils, immature dendritic cells, macrophages, mast cells, myeloid-derived suppressor cells, monocytes, natural killer cells, natural killer T cells, neutrophils, plasmacytoid dendritic cells, dendritic cells, myeloid dendritic cells, T cells.
Aran et al.<br>xCell, TIminer	Activated dendritic cells, adipocytes, alternatively activated macrophages (M2), astrocytes, B cells, basophils, classically activated macrophages (M1), $CD4^+$ central memory T cells, $CD4^+$ effector memory T cells, $CD4^+$ memory T cells, $CD4^+$ naïve T cells, $CD4^+$ T cells, $CD8^+$ central memory T cells, $CD8^+$ effector memory T cells, $CD8^+$ naïve T cells, $CD8^+$ T cells, chondrocytes, class-switched memory B cells, common lymphoid progenitors, common myeloid progenitors, conventional dendritic cells, dendritic cells, endothelial cells, eosinophils, epithelial cells, erythrocytes, fibroblasts, gamma-delta T cells, granulocytes, macrophage progenitors, hematopoietic stem cells, hepatocytes, immature dendritic cells, keratinocytes, lymphatic endothelial cells, macrophages, mast cells, megakaryocyte–erythroid progenitors, megakaryocytes, melanocytes, memory B cells, mesangial cells, mesenchymal stem cells, microvascular endothelial cells, monocytes, multipotent progenitors, myocytes, naïve B cells, natural killer cells, natural killer T cells, neurons, neutrophils, osteoblasts, pericytes, plasma cells, plasmacytoid dendritic cells, platelets, preadipocytes, pro B cells, regulatory T cells, sebocytes, skeletal muscle cells, smooth muscle cells, type 1 helper T cells, type 2 helper T cells.
Becht et al.<br>MCP-counter	T cells, $CD8^+$ T cells, cytotoxic lymphocytes, natural killer cells, B cells, cells from monocytic lineage, myeloid dendritic cells, neutrophils, endothelial cells, fibroblasts.
Charoentong et al.<br>TIminer	Activated B cells, activated $CD4^+$ T cells, activated $CD8^+$ T cells, central memory $CD4^+$ T cells, central memory $CD8^+$ T cells, effector memory $CD4^+$ T cells, effector memory $CD8^+$ T cells, gamma-delta T cells, immature B cells, memory B cells, regulatory T cells, T follicular helper cells, type 1 helper T cells, type 17 helper T cells, type 2 helper T cells, activated dendritic cells, $CD56^{bright}$ natural killer cells, $CD56^{dim}$ natural killer cells, eosinophils, immature dendritic cells, macrophages, mast cells, myeloid-derived suppressor cells, monocytes, natural killer cells, natural killer T cells, neutrophils, plasmacytoid dendritic cells.
Finotello et al.<br>quanTIseq	B cells, monocytes, classically activated (M1) macrophages, alternatively activated (M2) macrophages, neutrophils, natural killer cells, $CD4^+$ T cells, $CD8^+$ T cells, $CD4^+$ regulatory T cells, dendritic cells, other uncharacterized cells.
Li et al.<br>TIMER	Dendritic cells, macrophages, neutrophils, $CD8^+$ T cells, $CD4^+$ T cells, B cells.
Racle et al.<br>EPIC (blood)	B cells, $CD4^+$ T cells, $CD8^+$ T cells, monocytes, neutrophils, natural killer cells, other uncharacterized cells.
Racle et al.<br>EPIC (tumor)	B cells, $CD4^+$ T cells, $CD8^+$ T cells, macrophages, natural killer cells, endothelial cells, cancer-associated fibroblasts, other uncharacterized cells.
